# Supplementary material for: Content-rich biological network constructed by mining PubMed abstracts
Source: BMC Bioinformatics. 2004 Oct 8;5:147. doi: 10.1186/1471-2105-5-147 (PMC528731; doi:10.1186/1471-2105-5-147)
Supplement: Additional File 2 — The original results of the above study (non-essential files are deleted to keep the file size under the limit set by BMC bioinformatics). [file 1471-2105-5-147-S2.bz2 › chilibotAdditionalFile2/dip10/245ID8969169E801/html/IFNAR1_IFNAR2.html]

 


 **IFNAR1** and **IFNAR2** 
  
Found 94 abstracts in PubMed, retrieved 10.  
 

 What does Google say? 
 PDF only 
| .edu only 

---

**Interactive relationship** (e.g. stimulation, inhibition, etc)

**Neutral relationship**- The interaction of IFNs with their heterodimeric type I IFN receptor comprised of  **IFNAR1**  and  **IFNAR2**  is a first step to inducing biological actions.  Ref: 12542398 Biochem J, 2003
- Recombinant proteins corresponding to the cytoplasmic domains of the receptor subunits and the central core region of Stat2 were partially purified and used in affinity precipitation experiments to demonstrate that Stat2 binds more avidly to  **IFNaR2**  than to phosphorylated  **IFNaR1** .  Ref: 12220192 Biochemistry, 2002
- The interferon alpha receptor  [ **IFNAR1** ]  is composed of two subunits  **IFNaR1**  and  **IFNaR2** .  Ref: 12220192 Biochemistry, 2002
- Affinity precipitation experiments involving all three proteins Stat2, phosphorylated  **IFNaR1** , and  **IFNaR2**  indicated that the Stat2 receptor interactions are independent of one another.  Ref: 12220192 Biochemistry, 2002
- Stat2 bound  **IFNaR2**  K d = 45 nM approximately 6 fold stronger than it bound tyrosine 466 phosphorylated  **IFNaR1**  K d = 245 nM.  Ref: 12220192 Biochemistry, 2002
- The class II cytokine receptor CIICR genes Il10r2 and  **Ifnar1**  are localized on mouse Chr 16 in a cluster that also contains the CIICR genes  **Ifnar2**  and Ifngr2.  Ref: 12584606 Mamm Genome, 2003

**Non-interactive relationship** (e.g. studied together, co-existance, homology, etc.)

- Liver biopsy specimens from 41 chronic hepatitis C patients subsequently treated with IFN were immunostained with  **IFNAR1**  and  **IFNAR2**  antibodies, and also analyzed for both receptor subunit mRNA levels, using competitive polymerase chain reaction.  Ref: 12699848 Hepatol Res< MedlineTA>Hepatol Res, 2003
- The focus of this study is to determine both mRNA and protein expression levels of the type I interferon IFN receptor subunits, IFNalpha receptor  **IFNAR1**  and IFNalpha beta receptor  **IFNAR2** , in the liver, and to assess which quantification method is most useful in predicting response to IFN in chronic hepatitis C patients.  Ref: 12699848 Hepatol Res< MedlineTA>Hepatol Res, 2003
- Hepatic expression of  **IFNAR1**  r=0.45, P=0.012 or  **IFNAR2**  mRNA r=0.46, P=0.009 was weakly correlated with their protein expression in hepatocytes.  Ref: 12699848 Hepatol Res< MedlineTA>Hepatol Res, 2003
- To assess whether hepatitis C virus infection HCV affects type I interferon IFN receptor expression, the expression levels of two subunits of this receptor,  **IFNAR1**  and  **IFNAR2** , in peripheral blood mononuclear cells PBMCs were determined by flow cytometric assay in 68 anti HCV antibody positive patients 47 positive for HCV RNA and 21 negative for HCV RNA and 14 healthy controls.  Ref: 12787799 Hepatol Res< MedlineTA>Hepatol Res, 2003
- In the type I interferon receptor  [ **IFNAR2** ] , Tyk2 associates with the  **IFNAR1**  receptor subunit and positively influences ligand binding to the receptor complex.  Ref: 12554654 EMBO J, 2003
- For this report, we tested the pharmacogenomic effects of eight polymorphisms in the interferon receptor genes  **IFNAR1**  and  **IFNAR2**  in a group of 147 patients undergoing open label IFNbeta therapy.  Ref: 12618863 Genes Immun, 2003
- The potent antiviral and antiproliferative activities of human type I interferons IFNs are mediated by a single receptor comprising two subunits,  **IFNAR1**  and  **IFNAR2** .  Ref: 12842042 Structure Camb , 2003
- Immunostaining of  **IFNAR1**  and  **IFNAR2**  was observed in the cell membrane and cytoplasm of hepatocytes in all liver specimens.  Ref: 12699848 Hepatol Res< MedlineTA>Hepatol Res, 2003
- In multiple regression analysis, the presence of HCV RNA in serum was independently associated with the expression of both  **IFNAR1**  and  **IFNAR2**  in PBMCs P=0.007 for  **IFNAR1**  and P=0.003 for  **IFNAR2** .  Ref: 12787799 Hepatol Res< MedlineTA>Hepatol Res, 2003
- The percentages of  **IFNAR1**  and  **IFNAR2**  expressing cells were significantly higher in HCV RNA positive patients than in HCV RNA negative patients or healthy controls.  Ref: 12787799 Hepatol Res< MedlineTA>Hepatol Res, 2003
- In addition, we sought for genetic polymorphism in the promoter region of the interferon alpha  [ **IFNAR1** ]  beta receptor 2 gene  **IFNAR2** , and found single nucleotide polymorphisms SNPs at 134 and 75.  Ref: 12697242 Hepatol Res< MedlineTA>Hepatol Res, 2003
- To explore the function of the Stat2  **IFNaR2**  interaction and its possible relationship to the SH2 dependent docking of Stat2 to phosphorylated  **IFNaR1** , the affinity of Stat2 for each of the receptor subunits was determined.  Ref: 12220192 Biochemistry, 2002
- The labeling indexes of  **IFNAR1**  136.7 94.1 vs. 77.4 76.8, P=0.032 and  **IFNAR2**  153.1 80.2 vs. 87.2 75.5, P=0.011 in liver specimens were significantly higher in sustained virologic responders n=17 than in non sustained virologic responders n=24, while mRNA levels of either receptor subunit did not differ between response groups.  Ref: 12699848 Hepatol Res< MedlineTA>Hepatol Res, 2002
